# A Retrospective Analysis of Foreign Body Ingestions Among the Pediatric Age Group in a Tertiary Care Hospital in Jeddah, Saudi Arabia

**DOI:** 10.7759/cureus.48113

**Published:** 2023-11-01

**Authors:** Rahaf L Abudungor, Deema O Arif, Yasmeen S Alsulaiman, Dana A Alrabghi, Ahmed F Jarb, Lojien M Algari

**Affiliations:** 1 Faculty of Medicine, Fakeeh College for Medical Sciences, Jeddah, SAU; 2 Faculty of Medicine, Ibn Sina National College for Medical Studies, Jeddah, SAU; 3 Department of Pediatrics, Dr. Soliman Fakeeh Hospital, Jeddah, SAU

**Keywords:** button battery, coin, pediatrics, foreign body, ingestion

## Abstract

Background: Foreign body ingestion (FBI) among the pediatric age group is considered a major clinical problem that can cause life-threatening complications, as it can obstruct the airway due to poor/immature airway protection reflexes.

Objective: In this study, we aimed to retrospectively describe the epidemiology, clinical characteristics, and outcomes of FBI among the pediatric age group in Dr. Soliman Fakeeh Hospital, Jeddah, Saudi Arabia.

Methods: We conducted a retrospective study of pediatric patients (0-14 years) presenting to a tertiary care hospital in Jeddah, Saudi Arabia, from January 2019 to October 2022. The study reviewed records of patients with FBI in the emergency department. Data collection included age, gender, comorbidities, foreign body (FB) type, anatomical location, presenting symptoms, time to emergency room (ER) presentation, need for endoscopy, and complications. We performed a statistical analysis using the Statistical Package for Social Sciences (SPSS) 25 (IBM SPSS Statistics, Armonk, NY), where p<0.05 was considered statistically significant.

Results: We identified 244 FBI cases, with most cases being male (62.7%). The most common site of FB impaction was the stomach (38.9%), followed by the upper esophagus (29.1%). Clinical presentation was variable, with 20.5% of cases experiencing vomiting, 13.5% experiencing drooling, and 9.4% experiencing dysphagia. Out of 244 cases, 132 (54.1%) were referred to gastroenterology for urgent FB removal by endoscopy. A total of 186 cases (76.2%) did not have complications, whereas 3.6% had serious sequela. The association between age and FBI was statistically significant (p=0.00), whereas there was no association between gender and FBI.

Conclusion: Our results showed that FB ingestion was prevalent among children at our tertiary care hospital, with urgent endoscopy being the most common removal procedure. Early detection and immediate presentation to the emergency room are crucial for preventing complications. Common FBI included coins and batteries, with most incidents in 1-3-year-old males. Parents should be aware of the dangers of FBI and implement preventive measures to reduce its incidence.

## Introduction

Foreign body ingestion (FBI) among the pediatric age group is considered a major clinical problem that can cause life-threatening complications, as it can obstruct the airway due to poor/immature airway protection reflexes [1]. Some ingested foreign bodies (FBs) can pass through the gastrointestinal (GI) tract spontaneously without any complications. However, FBs can sometimes cause significant damage, especially when stuck in the esophagus, which is the most common site of impaction [2,3].

Batteries and coins are considered the most frequently ingested FBs that are accidentally or intentionally swallowed [4]. Button batteries generate electrical current and produce sodium hydroxide, which can cause liquefaction necrosis of the mucosa [5,6]. Therefore, esophageal damage can occur as rapidly as 2.5 hours after ingestion, and perforation can occur within six hours after ingestion [7].

The mortality rate from the aspiration of FB is approximately 24%, which has been decreasing due to recent advances in bronchoscope techniques and advances in critical care [8]. Presentation differs depending on the nature of the ingested FB, including its size and location. Possible symptoms of FBI include coughing, difficulty breathing, vomiting, and choking [9]. ﻿For suspected FBI, the standard radiologic workup is a chest X-ray, which can show the trachea and stomach in both lateral and anterior-posterior views. The double rim sign and/or the step sign is commonly seen in button battery ingestion [3].

A study conducted in 2018 in Cheongju, Korea, found that 80%-90% of FBs in the GI tract were passed spontaneously without any complication, 10%-20% were removed endoscopically, and 1% required open surgery secondary to complications [10]. A 2008-2016 retrospective study done in the Netherlands demonstrated serious complications from button battery injury, including mortality in 1% of cases, esophageal-tracheal fistula in 5% of cases, stenosis in 5% of cases, vocal cord paralysis in 1% of cases, and reintubation due to dyspnea and stridor in 1% of cases [2].

A 2013-2017 study conducted in an academic hospital in Jeddah, Saudi Arabia, analyzed 69 cases of FBI, in which the most commonly ingested FBs were coins, followed by batteries. In their study, males (56%) had a slightly higher rate of FBI than females (54%). Forty-five percent of their patients were symptomatic, mostly with vomiting, salivation, choking, dysphagia, and pain in the neck or abdomen. Thirty-two percent of cases were asymptomatic, and there was no documentation of symptoms in the remaining 23% [4]. A 2018 cross-sectional study in the Eastern Province of Saudi Arabia found that the majority of FBI cases were under five years old, with button batteries being associated with the highest mortality rate [1].

To the best of our knowledge, few studies have been published regarding FBI in Jeddah, Saudi Arabia. Therefore, we aimed to retrospectively describe the epidemiology, clinical characteristics, and outcomes of FBI among the pediatric age group in Dr. Soliman Fakeeh Hospital, Jeddah, Saudi Arabia.

## Materials and methods

After receiving ethical approval from the Institutional Review Board of Dr. Soliman Fakeeh Hospital, a tertiary care center in Jeddah, Saudi Arabia, we conducted a retrospective cohort study among the pediatric age group (0-14 years). We submitted a request for the Medical Records Committee to provide us with medical record numbers (MRNs) of patients who presented to the emergency department with FBI for the past three years (January 2019 to October 2022).

We received a total of 244 patient MRNs, for which a data collection sheet was developed prior to collection, which included the patient’s age, gender, presence of comorbidities, type of FB, anatomical location of FB, presenting symptom, time until presentation to the emergency room (ER), need for endoscopy, and presence of any complications following FBI. We included all 244 patients in our study. Informed consent was not taken, as the patients’ information was not identifiable.

Data collection was done by using the YASASII Healthcare Information System (Kameda Infologics Pvt. Ltd, Thiruvananthapuram, India). All statistical analyses were performed using ﻿the Statistical Package for Social Sciences (SPSS) 25 (IBM SPSS Statistics, Armonk, NY). Analyses were done according to the type of variables, including descriptive statistics, continuous variables (means and standard deviations), and categorical variables (frequencies and percentages). We used the chi-square test to assess the association between different variables, with p<0.05 considered statistically significant.

## Results

In the current retrospective study, a total of 244 cases of FBI were identified from the period 2019 to 2022, with 62.7% (n=153) being male cases and 37.3% (n=91) being female cases. In addition, 47% (n=116) of cases were 1-3 years of age, with a mean age of 3.75±2.72 years (Table 1).

**Table 1:** Demographic data of the study participants.

Demographic data		Male	Female	Total (n)
Age	Infants (<1 year)	9	13	22
	Toddler (1-3 years)	62	54	116
	Preschool (3-6 years)	52	16	68
	School age (6-13 years)	30	8	38
Total (n)		153	91	244
Medical history	Medically free	134	84	218
	Bronchial asthma	8	3	11
	Autism	1	0	1
	Esophageal stricture	1	0	1
	Global developmental delay	1	0	1
	Other comorbidities	8	4	12
Total (n)		153	91	244

**Figure 1:**
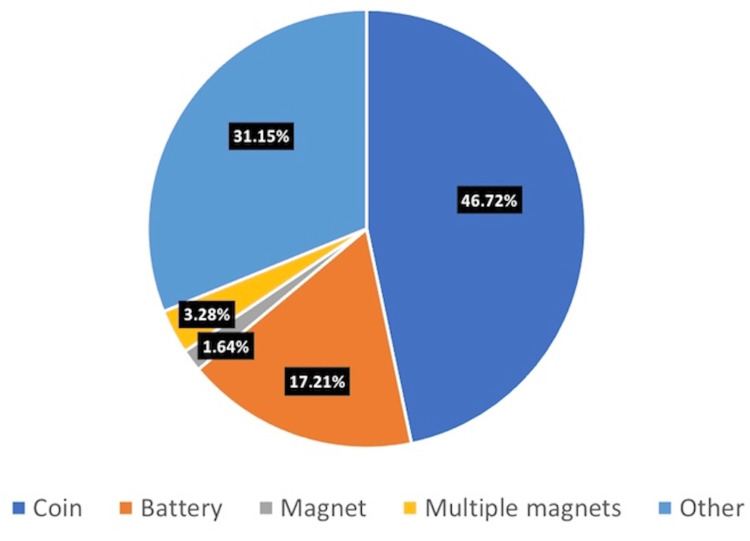
Type of foreign body ingestion.

Almost 46.7% (n=114) of the patients presented with coin ingestion, 17.2% (n=42) presented with battery ingestion, and 3.3% (n=8) presented with multiple magnet ingestion. Patients who presented with other FBI such as rings, necklaces, buckles, beads, earrings, glass, soap, needles, pen covers, plastic, rubber material, screws, fish bones, and food impaction accounted for 31.1% (n=76) of cases (Figure 1).

**Figure 2:**
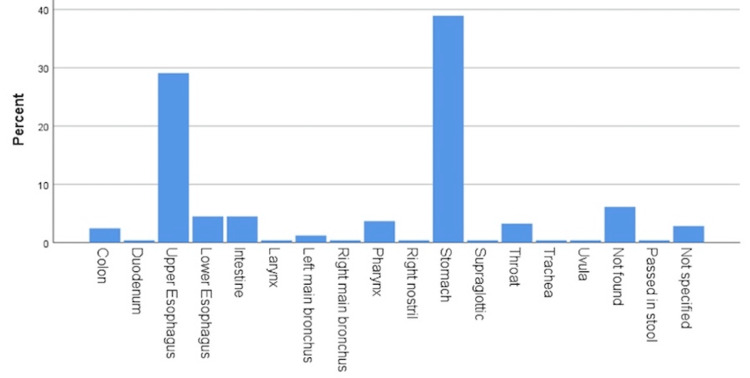
Site of foreign body.

The most common site of FB impaction was the stomach (38.9%; n=95), followed by the upper esophagus (29.1%; n=71) (Figure 2).

Clinical presentation was variable among our patients. Approximately 20.5% (n=50) presented with vomiting, 13.5% (n=33) presented with drooling, and 9.4% (n=23) presented with dysphagia (Table 2).

**Table 2:** Clinical presentation of foreign body ingestion.

Clinical presentation	Percentage
Vomiting	20.5%
Drooling	13.5%
Dysphagia	9.4%
Choking	4.1%
Abdominal pain	7%
Chest pain	3.3%
Cough	4.9%

Out of 244 cases of FBI, 132 (54.1%) patients were referred to gastroenterology for urgent FB removal by endoscopy. In contrast, 91 patients (37.3%) required no intervention and only observed stool for FB passage. The remaining 21 patients (8.5%) required either ear, nose, and throat (ENT) or cardiothoracic referral.

A total of 186 cases did not have any complications (76.2%). Nine patients (3.6%) had serious sequelae, including aspiration pneumonia, eosinophilic esophagitis, esophageal stricture, esophageal varices, extensive burn of the esophagus, erosion, and severe esophageal ulcers. Aspiration pneumonia and eosinophilic esophagitis were noticed in patients who were presented with food impaction, while other sequelae were noticed in patients with battery ingestion.

A total of 49 patients (20%) did not follow up after their initial presentation to the emergency room or were discharged against medical advice.

In our study, the association between gender and FBI was not statistically significant (p=0.19). On the other hand, the association between age and FBI was statistically significant (p=0.00) (Table 3).

**Table 3:** Statistical analysis of age and types of foreign body ingestion.

Type of foreign body		Coin (n)	Battery (n)	Magnet (n)	Others (n)	Total (n)	p=0.00
Age (years)	Infants (<1)	2	9	0	11	22	
	Toddler (1-3)	49	24	3	40	116	
	Preschool (3-6)	45	7	7	9	68	
	School age (6-13)	18	2	1	16	38	
Total		114	42	12	76	244	

The association between time until presentation to the emergency room and complications of FBI was not statistically significant (p=1.00). In contrast, the association between time until urgent endoscopy and the presence of complications was statistically significant (p=0.00).

## Discussion

Our results revealed that the majority of FBI cases occurred in males 62.7% (n=152). A previous study done in Ilorin showed a male-to-female ratio of 1.6:1 [11]. Another study conducted in China showed that the overall incidence of FBI was significantly higher among males (69.1%) [12], which reflects how gender is an influencing factor of FBI among the pediatric age group. This influence could be due to differences in physiological development, such as the development of mastication, laryngeal protection, and swallowing coordination [12]. However, the association between gender and FBI in our study was not statistically significant (p=0.19).

The association between age and FBI was statistically significant in our study, with the 1-3-year-old age group accounting for 47% (n=116) (p=0.00) of cases. This result agreed with a previous study done in 2017, which reported that age was one of the most influencing factors in FBI [13]. In our study, coin ingestion was the most common FBI type, representing 46.7% (n=111) of cases; 17.2% (n=40) of cases presented with battery ingestion, which was similar to studies done in Saudi Arabia in 2018 and 2023 [4,14].

As shown in Table 2, the clinical presentation was variable; 20.5% (n=50) presented with vomiting, 13.5% (n=33) with drooling, and 9.4% (n=23) with dysphagia. By comparison, in another study, 45% of FBI patients were symptomatic, mostly with drooling, vomiting, dysphagia, and pain in the neck or abdomen [4].

The most commonly used procedure for FB removal was urgent endoscopy, accounting for 54.1% of all cases in our study, which was similar to another study [4]. In addition, in a study done in Makkah, 95.4% of FBI patients required urgent endoscopic removal of the FB [14]. Furthermore, in a study done in Kurdistan, out of 20 patients, 13 required endoscopic intervention [3]. These reports highlight the importance of endoscopic procedures in preventing major complications associated with FBI [15].

Of our patients, 37.3% required no intervention, which may be due to the size and site of the FB that was ingested. According to a 2023 study done in Turkey, cases of a blunt object or coin in the stomach and an asymptomatic patient were followed up with radiographic images at 1-2-week intervals and given laxatives as needed; elective endoscopy was booked if the FB was not passed within 2-4 weeks [15].

Out of all 244 cases, nine patients (3.6%) had serious sequelae following FBI, which may be related to unwitnessed FBI and late presentation to the hospital. A 2023 case report was published in Riyadh of a 25-month-old female who presented to the emergency department with upper GI bleeding and low hemoglobin, who eventually died during resuscitation due to unwitnessed FBI [16]. In another study, a patient who presented two weeks after button battery ingestion passed away due to uncontrolled bleeding caused by esophageal-aortal fistula [2]. Therefore, it is important to suspect FBI in pediatric patients who present to the emergency department with GI bleeding, as they can ingest foreign objects without someone else witnessing it. It is also very crucial to prevent FBI and to teach parents and caregivers about childhood safety.

This study did have some limitations. For example, the data were only obtained from a single tertiary care hospital in Jeddah, Saudi Arabia; additional data from various healthcare facilities/hospitals could have provided us with more information regarding FBI. As Dr. Soliman Fakeeh Hospital began utilizing the YASASII Healthcare Information System, which is the electronic health record in 2019, information prior to that time was not retrievable. This information could have provided us with more data for further comparison.

## Conclusions

Based on the results of this study, FBI is prevalent among children in a tertiary care hospital in Jeddah, Saudi Arabia. Urgent endoscopy was the most commonly used procedure for FB removal. The early detection of FBI and immediate presentation to the emergency room are crucial in preventing potentially serious complications. Our results indicate that the most common FBs ingested were coins and batteries. Therefore, parents should take extra precautions with these items, especially around preschool toddlers, as our study found that most incidents were in the 1-3-year-old age group. We recommend that awareness should be raised among parents regarding the dangers of FBI, as well as preventive measures that can be taken to reduce the incidence of FBI.
